# Human papillomavirus (HPV) testing on self-collected specimens: perceptions among HIV positive women attending rural and urban clinics in South Africa

**DOI:** 10.11604/pamj.2014.17.189.3454

**Published:** 2014-03-11

**Authors:** Kay Mahomed, Denise Evans, Celeste Sauls, Karin Richter, Jennifer Smith, Cindy Firnhaber

**Affiliations:** 1 Right to Care, Johannesburg, South Africa; 2 Health Economics and Epidemiology Research Office, Department of Internal Medicine, School of Clinical Medicine, University of the Witwatersrand, Johannesburg, South Africa; 3 Department of Medical Virology, University of Pretoria, National Health Laboratory Service, Pretoria, South Africa; 4 Department of Epidemiology, Gillings School of Global Public Health, University of North Carolina, United States of America; 5 Clinical HIV Research Unit, Department of Internal Medicine, Faculty of Health Sciences, University of the Witwatersrand, Johannesburg, South Africa

**Keywords:** HIV-positive women, self-collection, human papillomavirus, acceptability, cervical cancer, resource-limited setting, South Africa

## Abstract

**Introduction:**

Cervical cancer is the most common cancer among women in Sub-Saharan Africa. Cervical cancer is treatable if detected timeously, yet only 20% of South African women have ever been for a Pap smear in their lifetime due to limited access to screening, transport or child care responsibilities.

**Objective:**

To evaluate the acceptability of self-collection for cervical cancer screening. We aimed to identify which self-collection device women prefer and if they would consider using them for routine cervical cancer screening.

**Methods:**

HIV-positive women (>18 years) from urban and rural HIV clinics were interviewed following an education session on HIV, human papillomavirus (HPV) and cervical cancer. Participants were shown three self-collection devices; (i) an Evalyn cervical brush, (ii) a Delphilavager and (iii) a tampon-like plastic wand before completing a short questionnaire.

**Results:**

A total of 106 women from the urban (n = 52) and rural (n = 54) clinic were interviewed. Overall 51% of women preferred the cervical brush, while fewer women preferred the tampon-like plastic wand (31%) or lavage sampler (18%). More than 75% of women from the rural site preferred the cervical brush, compared to 22% from the urban site (p < 0.001). Women from the urban clinic preferred the tampon-like plastic wand (45%) and then the lavage sampler (33%), as compared to women from the rural clinic (19% and 4%, respectively).

**Conclusion:**

Women from urban or rural settings had different preferences for the various self-collection devices. Patient self-collection with HPV testing may be an acceptable way to improve coverage to cervical cancer screening in high risk HIV-seropositive women.

## Introduction

Cervical cancer is the most prevalent cancer in HIV-infected women in sub-Saharan Africa and the leading cancer-related cause of death among women [1]. Approximately 80% of cases worldwide occur in less developed countries, where cervical cancer accounts for 15% of female cancers compared to 3.6% in developed countries. An estimated 70,700 annual cases occur each year in sub-Saharan Africa [2]. In females, the lifetime risk of dying from cancer in Africa is almost double the risk in developed countries [2]. Most cases of women with cervical cancer in sub-Saharan Africa present at advanced stages of the disease, given that treatment is generally not available. Cervical cancer is detectable and preventable through cervical screening for pre-cancerous lesions (Papanicolaou or Pap smear); however this is often not effective in most developing countries where adequate health infrastructure, human and financial resources are not available [3].

Oncogenic types of human papillomavirus (HPV) are the central cause of cervical cancer and its precursor lesions.[4] HPV16, 18, 45 and 35 are the most common HPV types in sub-Saharan African women with invasive cervical cancer (ICC)[5]. In a study amongst women with ICC (167 in Ghana, 192 in Nigeria and 300 in South Africa), HPV-positivity rate in ICC cases was 90% (515/570) with HPV16 (51%), HPV18 (17%), HPV35 (9%), HPV45 (7%), HPV33 (4%) and HPV52 (2%) being the most common HPV types detected.[6] Women with persistent HPV infections are at high risk of cervical lesions and cancer [7].

HPV infections are more common in HIV-positive women and the prevalence of single and multiple HPV infections is higher among HIV-positive women than HIV-negative women.[6] In HIV-infected woman, there is higher prevalence of HPV infection (64% compared to 28% in HIV-negative women), with a significant increased risk of cervical abnormalities[8]. In South Africa HIV-positive women have almost a 4-5 times greater incidence of cervical cancer.

Women living in resource-limited countries are especially at risk due to poor access to cervical cancer screening and treatment [9]. Invasive cervical cancer also tends to present earlier in HIV infected than HIV negative women [10].

Precursor lesions of cervical cancer are highly amenable to treatment and therefore identifying effective screening methods allows for the prevention of invasive cervical cancer and associated morbidity and mortality. Screening for cervical cancer in women 30 years and older is well established in resourced countries where the Pap test with the HPV test forms the hallmark of screening[11]. In many resourced countries, however, screening for cervical cancer is still far from ideal. Coverage is often worse in resource-limited settings because of competing healthcare priorities. A coverage around 50% prevails in some countries, and few have reached the target of 80% or more [11]. Reasons include lack of trained providers and the infrastructure necessary to perform testing, inconvenient clinic hours and women not being comfortable with having a Pap smear done.

HPV screening through patient self-collection has been shown to have a high sensitivity and specificity for the detection of high risk HPV types which can lead to cervical cancer. Sensitivity and specificity for detection of cervical intraepithelial neoplasia grade 2 or worse (CIN2+)was 87.5% and 77.2% for self-sampling using a cervical brush compared to 96.8% and 79.7% for the direct test (p < 0.001), respectively.[12] HPV testing has been shown to be an effective approach to cervical cancer screening. A self-collection test or self-test, collects HPV using a device such as a cervical brush, vaginal swab or vaginal lavage[13]. These devices can be used at home and returned to the clinic or laboratory by courier or mail to increase cervical screening rates [13]. Self-collection sampling for HPV testing could be a potential alternative to Pap smear test, provided that women who test positive by any method get timely follow-up and care.[14] Self-testing could be helpful in several scenarios in South Africa. In rural areas where there may be no infrastructure to perform Pap smears, community health care workers in mobile vans could educate and distribute the HPV testing devices for self-collection at home or within the community center/van. Women can be contacted or return for results and if positive be referred to a clinic for screening by Pap smear or visual inspection with acetic acid and treatment with cryotherapy, depending on the standard of care. Another possibility is while women are waiting in the clinic for other health care needs (i.e. medication) they could self-test and then be contacted or return for their results. If the result is HPV positive the patient can be referred for further evaluation.

Studies have assessed the acceptability and usability of self-collected sampling for HPV testing in resource-developed settings and some resource-limited settings such as Mexico and China. however there is limited data on the acceptability of HPV self-collection in South African HIV-infected women, a population that are most likely to benefit if implemented [12–15]. We therefore aimed to identify which self-collection device women prefer and if they would be willing to use it for routine cervical cancer screening.

## Methods

### Study design and population

Questionnaires assessing attitudes and preferences were conducted among HIV positive patients attending two HIV treatment clinics; ThembaLethu Clinic, Johannesburg, South Africa (urban) and Topsy Clinic, Mpumalanga, South Africa (rural). ThembaLethu Clinic is one of the largest clinics providing ART in South Africa. It is an urban clinic, located at the Helen Joseph Hospital in Johannesburg. The clinic is an accredited public sector Comprehensive Care, Management and Treatment (CCMT) site for HIV-positive patients. Firnhaber and colleagues reported that 95% of women tested at this clinic (n = 148) had HPV DNA with 83% having 1 or more HPV oncogenic types [16].

The Topsy Clinic is located in rural Southern Mpumalanga. This province has one of the higher provincial prevalence rates of HIV, being 15.4% in 2008, and with pregnant women at 32% in 2007. The Topsy Foundation partners with rural communities, empowering people infected with, and affected by HIV and AIDS, through medical care, social support and skills development.

HIV-positive adult (>18 years of age) women, willing to participate in the study and provide written informed consent, were enrolled consecutively in the study. Participants were English speaking, between 20 and 65 years of age and were not pregnant.

Ethics approval was obtained from the University of the Witwatersrand (protocol number HREC M121051).

### Study procedure

**Figure 1:**
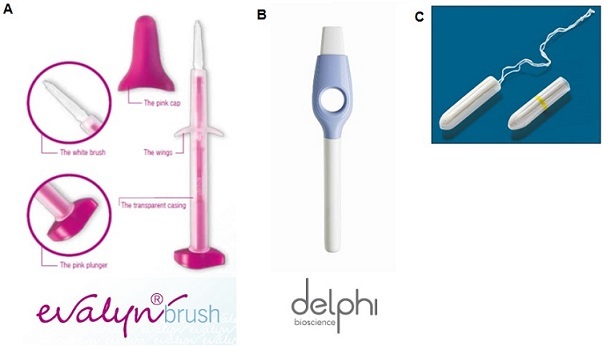
A): The Evalyn cervical brush, is a brush that is insert into the vagina and is turned around 5 times to collect cells; B): The Delphi lavager, releases liquid into the vagina and collects fluid back into the device to collect cells; C): the Fournier cervical self-sampling device is a tampon-like plastic wand that is also inserted into the vagina and turned around to collect cells

After obtaining informed consent, women were offered an educational session on HIV, HPV and cervical cancer, presented in English, by the attending clinician. This was followed by a self-administered questionnaire comprising of 15 questions. Participants were shown three different self-collection devices (Figure 1).

Three devices were used, each with an acceptable sensitivity (Se) and specificity (Sp) for the detection of high-grade cervical pre-cancer (CIN2+) using physician-collected cervical samples and cytology as the gold standard (cervical brush Se 83%, Sp 94%; lavager 81%, Sp 68%) or for the detection of oncogenic HPV (tampon-like plastic wand Se 94%, Sp 81%) [17–19] (Figure 1). The Evalyn cervical brush is a brush that women insert into the vagina and is turned around 5 times to collect cells. The Delphi lavager, is sterile, syringe-like device containing 5 milliliters of buffered saline. One operates it by plunging the handle, releasing the saline into the vagina, holding it down for 5 seconds, then releasing the handle, so that the device retrieves the fluid [20]. The user then plunges the lavage specimen into a prelabeled coded tube which can be returned to the clinic or laboratory. The Fournier cervical self-sampling device is a tampon-like plastic wand with an ejectable tip that is inserted into the vagina and turned around 15 to 20 times to collect cells [13].

The clinician briefly explained each device and how they work. Women could see and touch each device with the opportunity to ask questions before completing the questionnaire. The devices were presented in a different order to distribute the potential order effects equally, as described by Richman et al, 2011[13]. The order of the devices was randomly assigned according to patient enrolment. For example, study participants 1 through 10 were shown the cervical brush, lavager and then the tampon-like plastic wand (in order) while study participants 11 through 37 were shown the tampon-like plastic wand, cervical brush and then the lavager. The order in which the devices were presented was recorded for each study participant.

### Study Questionnaire

Participants were asked to complete an anonymous self-administered questionnaire in English. Questions included participant age and self-reported history of Pap-smear (ever and how recent). The questionnaire also included close-ended questions including if they would prefer self- collection devices over conventional Pap-smear and their preference and willingness to use the devices they were shown. In addition, participants were asked if they thought self-testing could be valuable over Pap-smear and if it could prevent cervical cancer.

### Statistical analysis

Participant questionnaires were captured in Excel and exported into SAS version 9.3 (SAS Institute, Inc., Cary, NC, USA). Patients were stratified into two groups: urban and rural. Groups were compared using Student t test (for normally distributed or parametric data) or Kruskal-Wallis (for not normally distributed or non-parametric data) for continuous variables and Chi-square (χ2) test or Fisher exact test for proportions, where appropriate. A p value < 0.05 was considered significant. Patient responses were summarized in Table 1.

**Table 1:** Summary of patient demographics and preferences of self-collection devices

Characteristics		Total	Urban	Rural	
		n = 106	n = 52	n = 54	p value
Age in years	Median (IQR)	40 (34 – 47)	39 (35 – 44)	40 (31 – 49)	0.77
Had a Pap smear before?	n,%	70/106 (66%)	37/52 (71%)	33/54 (61%)	0.28
Pap smear within the last 2 years	n,%	54/68 (79%)	30/38 (79%)	24/30 (80%)	0.91
Participant prefers self-testing over conventional Pap smear	n,%	99/105 (94%)	48/51 (94%)	51/54 (94%)	0.94
Participant believes that self-testing could prevent cervical cancer?	n,%	105/106 (99%)	51/52 (98%)	54/54 (100%)	0.31
Participant believes that there is value in self-testing	n,%	105/106 (99%)	51/52 (98%)	54/54 (100%)	0.31

IQR, interquartile range

## Results

A total of 106 HIV-positive women were recruited from the waiting rooms of two adult HIV government outpatient clinics: 52 from the urban clinic and 54 from the rural clinic. All women that were approached were willing to participate. Women were of similar age in both groups (median age of 40 years IQR 34-47; p = 0.767). More than 30% (n = 36/106) of women had never had a Pap smear, while 51% (n = 54/106) had one in the last 2 years. These numbers were similar in both groups (p = 0.190; p = 0.912) Table 1.

Overall 51% of women preferred the cervical brush, while fewer women indicated that they would rather use the tampon-like plastic wand (31%) or lavager (18%). There was a strong correlation between liking a device and being willing to use it (cervical brush r = 0.868; tampon-like plastic wand r = 0.921; lavager r = 0.928). All of the women indicated that they would be willing to perform a self-test for cervical cancer screening.

There were differences in the acceptability of different self-collection devices among women from urban or rural areas. More than 75% of women from the rural site preferred the cervical brush, compared to 22% from the urban site (p < 0.001). Women from the urban HIV clinic preferred the tampon-like plastic wand (45%) and then lavager (33%) compared to only a few women from the rural clinic (19% and 4%, respectively) Table 2.

**Table 2:** Preferences of self-collection devices, by site

	Total n= 106	Urban n = 52	Rural n = 54
Most preferred			
Cervical brush	52/102 (51%)	11/49 (22%)*	41/53 (77%)*
Tampon-like plastic wand	32/102 (31%)	22/49 (45%)	10/53 (19%)
Lavage sampler	18/102 (18%)	16/49 (33%)	2/53 (4%)
Least preferred			
Cervical brush	20/103 (19%)	13/50 (26%)	7/53 (13%)
Tampon-like plastic wand	47/103 (46%)	22/50 (44%)	25/53 (47%)
Lavage sampler	36/103 (35%)	15/50 (39%)	21/53 (49%)

* p < 0.001

## Discussion

In this cohort of rural and urban HIV-positive women there was a universal acceptance of self-collection as a potential way to improve coverage to cervical cancer screening in high risk HIV-seropositive women and complement conventional Pap smear testing. Urban women seemed to prefer the tampon-like plastic wand whereas rural women preferred the cervical brush. These results may have significant implications for future screening programs for cervical cancer in women with HIV.

Results from our study support those from other studies. In a study conducted in Mexico to assess the acceptability of HPV self-sampler, most patients reported being comfortable when using the self-test [15]. We assessed the acceptability of self-collected sampling but did not assess the accuracy of the self-collection devices, which is a weakness of our study. Numerous studies from resource-rich and resource-limited settings have reported the acceptability and usability of self-collected sampling for HPV testing.[12–14, 17–19] In a pooled analysis of over 13000 self-sampled HPV DNA tests from China, the sensitivity of self-HPV testing (Se 86.2% and Sp 80.7% for detecting CIN2+ and Se 86.1% and Sp 79.5% for detecting CIN3+) compared favourably with that of liquid-based cytology (Se 80.7% and Sp 94.0% for detecting CIN2+ and Se 89.0% and Sp 92.8% for detecting CIN3+) and was superior to the sensitivity of visual inspection with acetic acid (Se 50.3% and Sp 87.4% for detecting CIN2+ and Se 55.7% and Sp 86.9% for detecting CIN3+) [20]. In another study conducted in predominantly medically underserved, rural communities in Mexico the relative sensitivity of HPV testing was 3.4 times greater, but the specificity was lower. HPV testing detected over 4 times more invasive cancers than did cytology [21]. Such testing might be preferred for detecting CIN2+ or worse in low-resource settings where restricted infrastructure reduces the effectiveness of cytology screening programs [21]. By contrast, clinician-collected cytology performed with equal sensitivity to self- and clinician-collected samples for HPV in China and India and with higher specificity [22, 23]. Similarly, a study from Gambia showed that self-administered swabs showed a sensitivity of 63.9% and tampons showed a sensitivity of 72.4% compared to cervical cytobrush as the gold standard. When the acceptability and sensitivity were combined, self-administered swabs detected 61.9% and tampons detected 60.9% of the true positives [24].

Studies have shown that high-risk HPV testing on self-samples appears to be at least as, if not more, sensitive for CIN2+ as cytology on clinician-obtained cervical samples, though often less specific. Variations in clinical performance likely reflect the use of different combinations of collection devices and HPV tests [21]. However the specificity is decreased and in the HIV population in South Africa the high burden of HPV may not make HPV screening that effective as a screening tool [25, 26]. Screening test performance is also dependent on the operator and on locality-specific effectiveness of quality management (i.e. salaries, training, equipment, supplies, process measurements) [27]. It should also be noted that the devices are different, especially in terms of sampling different areas. For example the cervical brush sampling is directed towards the transformation zone, the area on the cervix where abnormal cells most commonly develop, while the lavage includes the whole cervical area [13]. Furthermore, among teenaged girls, the transformation zone lies on the cervix's outer surface, where it is more vulnerable to infection than it is in adult women [13]. Cervicovaginal lavage sampling may be superior to cervix-directed sampling for future HPV prevalence studies[13].

This study did not access barriers or factors that influenced women's perceptions of the different devices. Others have identified the most influential barriers were fear, lack of signs or symptoms of illness, husbands′ influence, cost, lack of time, being unable to read, and lack of trust in the medical community [15]. In a study conducted in Uganda, factors positively associated with women′s willingness to collect their own samples for HPV testing were agreement to let outreach workers deliver the necessary swab to their homes and willingness to undergo a pelvic examination if the sample was abnormal. Factors negatively associated were embarrassment at collecting the sample at home or lack of privacy and concern of not collecting the sample properly[28].

Studies have shown that HPV self-collected testing significantly improved the participation of women who did not routinely attend cervical cancer screening programs [29]. However improving the participation does not automatically translate into a decrease in cervical cancer. Screening programs require frequent repeats of the screening tests. They also require a functioning healthcare infrastructure, with laboratories for smear processing and interpretation, mechanisms for quality control, referral for colposcopy, treatment of precursors, and follow-up to detect failures of treatment. New technologies, specifically the development of liquid-based cytology, have improved the performance of cytology as a screening test, but do not obviate the infrastructural challenges posed to health systems by cytology-based screening programs [30]. It is however important to note that in a low-resource setting, a single round of HPV testing has been associated with a significant reduction in the numbers of advanced cervical cancers and deaths from cervical cancer [20].

Because it is known that under-screened women are at increased risk of cervical cancer, targeting non-attendees of the screening program could improve the effectiveness of cervical screening [31, 32]. Non-attendees are mostly from lower socioeconomic groups and self-testing has shown to facilitate access to cervical screening for women in low-resource areas [32]. In developed countries offering self-sampling has shown to be superior to a recall invitation for cytology in re-attracting original non-attendees into the screening program. This suggests that HPV self-sampling could be an additional strategy that can improve screening performance compared to current cytology-based call-recall programs [31]. However, the low compliance to follow-up amongst self-sampling reduces the effectiveness of this screening approach in non-attendees and should be carefully managed [32]. Of concern is the alarmingly high number of HIV-positive women who reported never having a Pap smear before (30%) or not having one in the past 2 years (49%) – results similar to those reported by Leyden and colleagues [33].

With the recent availability of commercial home-based self-collection tests in South Africa it will be important to assess the acceptability, accuracy and cost-effectiveness of self-sampling compared to cytology based screening in South Africa. It may also be interesting to assess the acceptability and uptake of these tests in urban and rural settings in South Africa in light of our study findings which show that urban women prefer the tampon-like plastic wand whereas rural women preferred the cervical brush.

## Conclusion

Our findings have implications not only for HPV DNA testing but also for other self-testing methods and suggestthat interventions may need to be context specific in order for them to be effective. Few studies have assessed the acceptability of self-testing for HPV in HIV-positive women, a high-risk population that are most likely to benefit from early detection and improved participation in routine cervical cancer screening. Future studies are needed to assess the acceptability, accuracy and cost-effectiveness of self-collection for HPV, Chlamydia, Gonorrhoea or STD′s in HIV-positive women.

Cervical cancer is a preventable cancer and a variety of screening mechanisms need to be evaluated to improve access in resource-limited areas and in areas of HIV prevalence such as Sub-Saharan Africa to reduce the disease burden and mortality of cervical cancer. Patient self-collection with HPV testing may be a way to improve coverage to cervical cancer screening in high risk HIV–seropositive women.
